# Effect of obesity on molecular characteristics of invasive breast tumors: gene expression analysis in a large cohort of female patients

**DOI:** 10.1186/s40608-016-0103-7

**Published:** 2016-04-29

**Authors:** Allyson L. Toro, Nicholas S. Costantino, Craig D. Shriver, Darrell L. Ellsworth, Rachel E. Ellsworth

**Affiliations:** Clinical Breast Care Project, Chan Soon-Shiong Institute of Molecular Medicine at Windber, 620 Seventh Street, Windber, PA 15963 USA; Clinical Breast Care Project, Murtha Cancer Center, Walter Reed National Military Medical Center and Uniformed Services University, 8901 Wisconsin Avenue, Bethesda, MD 20889 USA; Clinical Breast Care Project, Murtha Cancer Center, 620 Seventh Street, Windber, PA 15963 USA

**Keywords:** Breast cancer, Obesity, Gene expression

## Abstract

**Background:**

Obesity is a risk factor for breast cancer in postmenopausal women and is associated with decreased survival and less favorable clinical characteristics such as greater tumor burden, higher grade, and poor prognosis, regardless of menopausal status. Despite the negative impact of obesity on clinical outcome, molecular mechanisms through which excess adiposity influences breast cancer etiology are not well-defined.

**Methods:**

Affymetrix U133 2.0 gene expression data were generated for 405 primary breast tumors using RNA isolated from laser microdissected tissues. Patients were classified as normal-weight (BMI < 25), overweight (BMI 25–29.9) or obese (BMI ≥ 30). Statistical analysis was performed by ANOVA using Partek Genomics Suite version 6.6 using a false discovery rate <0.05 to define significance.

**Results:**

Obese patients were significantly more likely to be diagnosed ≥50 years or with African American ancestry compared to lean or overweight women. Pathological characteristics including tumor stage, size or grade, lymph node status, intrinsic subtype, and breast cancer mortality did not differ significantly between groups. No significant gene expression differences were detected by BMI in a non-stratified analysis which included all subtypes or within luminal B, HER2-enriched or basal-like subtypes. Within luminal A tumors, however, 44 probes representing 42 genes from pathways such as cell cycle, p53 and mTOR signaling, DNA repair, and transcriptional misregulation were differentially expressed.

**Conclusions:**

Identification of transcriptome differences in luminal A tumors from normal-weight compared to obese women suggests that obesity alters gene expression within ER+ tumor epithelial cells. Alterations of pathways involved in cell cycle control, tumorigenesis and metabolism may promote cellular proliferation and provide a molecular explanation for less favorable outcome of obese women with breast cancer. Targeted treatments, such as mTOR inhibitors, may allow for improved treatment and survival of obese women, especially African American women, who are more likely to be obese and suffer outcome disparities.

## Background

Data from the Centers for Disease Control and Prevention indicate that 68 % of adults in the United States (US) are overweight (25 ≤ BMI < 30 kg/m^2^) or obese (BMI ≥ 30 kg/m^2^) [1]. Obesity is associated with significantly higher all-cause mortality in the general population [2] and has been associated with type 2 diabetes, cardiovascular disease, asthma, osteoarthritis, and many types of cancer [3]. If obesity continues to escalate at current rates, total healthcare costs attributable to obesity-related care could reach >$860 billion by 2030 and account for 18 % of total healthcare expenditures in the US [4].

Obesity and weight gain between 20 and 50 years of age are significant risk factors for breast cancer [5] in postmenopausal women [6, 7], especially those not using hormone replacement therapy (HRT) [8]. Although the association between body mass index (BMI) and breast cancer subtype is unclear [9–15], obesity has been associated with less favorable pathological characteristics including advanced stage, larger tumor size and metastatic lymph node involvement [16–19]. In addition, meta-analyses have detected significant associations between obesity and both overall and breast-cancer specific survival [20, 21].

Given the increasing obesity epidemic in the United States and throughout the world, it is critical to understand how obesity influences breast cancer etiology. Poor prognosis may be attributable to co-morbid conditions, inadequate dosing with chemotherapeutic agents, or biological effects of excess adiposity including increased levels of estrogen, hyperinsulinemia, or chronic inflammation [22, 23]. To better understand relationships between the molecular landscape of tumor epithelial cells and adiposity, gene expression data was generated from 405 microdissected breast carcinomas and analyzed by BMI at the time of diagnosis.

## Methods

### Ethics, consent and permissions

All patients enrolled in the Clinical Breast Care Project met the following eligibility criteria: 1) adult over the age of 18 years, 2) mentally competent and willing to provide informed consent, and 3) presenting to the breast centers with evidence of breast disease. Tissue and blood samples were collected with approval from the Walter Reed National Military Medical Center Human Use Committee and Institutional Review Board. All subjects voluntarily agreed to participate and gave written informed consent.

### Specimen collection and characterization

Tissue was collected from patients undergoing surgical procedures, including lumpectomy or mastectomy. Within 5–15 min of surgical removal, breast tissue was taken on crushed, wet ice to the pathology laboratory where a licensed pathologist or pathologists’ assistant performed routine pathological analyses. Diagnosis of every specimen was conducted by a breast pathologist. Stage and grade were assigned using guidelines defined by the AJCC *Cancer Staging Manual* seventh edition [24] and the Nottingham Histologic Score [25, 26], respectively. Intrinsic subtype was determined using the BreastPRS as previously described [27].

### RNA isolation, amplification, aRNA labeling and hybridization

For each case, the breast pathologist identified tumor areas for laser microdissection from H&E stained slides. Two to five serial sections (8 μm thick) were cut, mounted on glass PEN foil slides (Leica Microsystems, Wetzlar, Germany), stained using the LCM staining kit (Applied Biosystems, Foster City, CA) and laser microdissected on an AS*LMD* laser microdissection system (Leica Microsystems, Wetzlar, Germany). Slide preparation, staining and cutting were performed within a 15 min period to preserve RNA integrity. RNA was then isolated using the RNAqueous-Micro kit (Applied Biosystems, Foster City, CA) and treated with DNase I to remove any contaminating genomic DNA. RNA integrity was assessed using the 2100 Bioanalyzer (Agilent Technologies, Santa Clara, CA), converted to biotin-labeled aRNA using two rounds of amplification with the MessageAmpII aRNA Amplification kit (Applied Biosystems, Foster City, CA), and the concentration and quality of the samples was measured with the NanoDrop 1000 (NanoDrop Products, Wilmington, DE) and 2100 Bioanalyzer. Hybridization with manufacturer provided hybridization controls, washing, staining and scanning of HG U133A 2.0 arrays (Affymetrix, Santa Clara, CA) were conducted according to manufacturer’s protocols [28].

### Analysis and statistics

For statistical analyses, BMI was not treated as a continuous variable; rather patients were classified as normal-weight (BMI < 25), overweight (BMI 25–29.9) or obese (BMI ≥ 30). Analysis of clinicopathological characteristics was performed using chi-square analysis (http://www.physics.csbsju.edu/stats/contingency_NROW_NCOLUMN_form.html) with *P <* 0.05 used to define significance.

Gene expression data were analyzed with Partek® Genomics Suite v 6.6 (Partek Incorporated). Probe set intensities were obtained by robust multi-array average background correction, quantile normalization, median polish summarization, and log_2_ transformation. Data integrity was then assessed by standard GeneChip® quality control parameters.

Principal component analysis (PCA) was performed using Partek® Genomics Suite v 6.6 to evaluate whether gene expression patterns effectively separated tumors by BMI. The three principal components accounting for the greatest portion of variability in gene expression were used to create a plot in order to visualize possible clustering by BMI groups. Analysis of variance (ANOVA) adjusted for age at diagnosis and self-described ethnicity was used to identify genes differentially expressed between BMI groups with a false discovery rate (FDR) <0.05. In the first analysis, all tumor specimens were included followed by subgroup analysis within intrinsic subtype groups (luminal A, luminal B, HER2-enriched, basal-like). Power analysis was performed using the MD Anderson Cancer Center sample size calculator (http://bioinformatics.mdanderson.org/MicroarraySampleSize/). Pathway enrichment was performed using the pathway analysis tool in Partek with an enrichment score of ≥2.0 defining significance.

## Results

### Patient characteristics

All patients were diagnosed with invasive breast cancer between 2001 and 2011. Obese women were significantly older at diagnosis (*P =* 0.009) and were significantly more likely to be African American (*P =* 0.012) than normal-weight women. Overweight women did not differ significantly for age at diagnosis or ancestry from either normal-weight or obese women. No pathological characteristics or patient outcomes differed significantly by BMI (Table 1).

**Table 1:** Clinical and pathological characteristics of 405 primary breast tumors evaluated by microarray analysis

	Normal-weight (*n* = 131)	Overweight (*n* = 132)	Obese (*n* = 142)	*P*-value
Age				0.031
<40 years	0.15	0.11	0.06	
40–49 years	0.27	0.20	0.22	
≥50 years	0.58	0.69	0.72	
Ethnicity				0.031
African American	0.17	0.19	0.32	
Asian	0.04	0.02	0.01	
Hispanic	0.02	0.02	0.01	
Other	0.01	0.02	0.01	
Non-Hispanic White	0.76	0.75	0.65	
Tumor Size				0.130
T1	0.59	0.56	0.50	
T2	0.35	0.34	0.45	
T3	0.06	0.10	0.05	
Tumor Grade				0.216
Well (Grade 1)	0.23	0.21	0.19	
Moderate (Grade 2)	0.42	0.35	0.32	
Poor (Grade 3)	0.35	0.44	0.49	
Intrinsic Subtype				0.560
Luminal A	0.52	0.48	0.54	
Luminal B	0.13	0.08	0.11	
HER2-enriched	0.11	0.16	0.08	
Basal-like	0.23	0.27	0.24	
Normal-like	0.01	0.01	0.03	
Lymph Node Status				0.447
Positive	0.36	0.40	0.44	
Negative	0.64	0.60	0.56	
TNM Stage				0.144
Stage I	0.45	0.40	0.31	
Stage II	0.41	0.42	0.48	
Stage III	0.12	0.12	0.18	
Stage IV	0.02	0.06	0.03	
Status^a^				0.929
Died of disease	0.08	0.07	0.07	
Died other causes	0.02	0.03	0.02	
Alive with disease	0.05	0.05	0.03	
Alive, disease-free	0.85	0.85	0.88	

^a^Patient status included died of disease if that patients died of metastatic breast cancer, and died other causes if a patient died from other health conditions. Patients Alive with disease were diagnosed with or have progressed to stage IV breast cancer while those Alive, disease-free have had no additional breast cancer-events since diagnosis and treatment of the original primary breast tumor

**Fig. 1:**
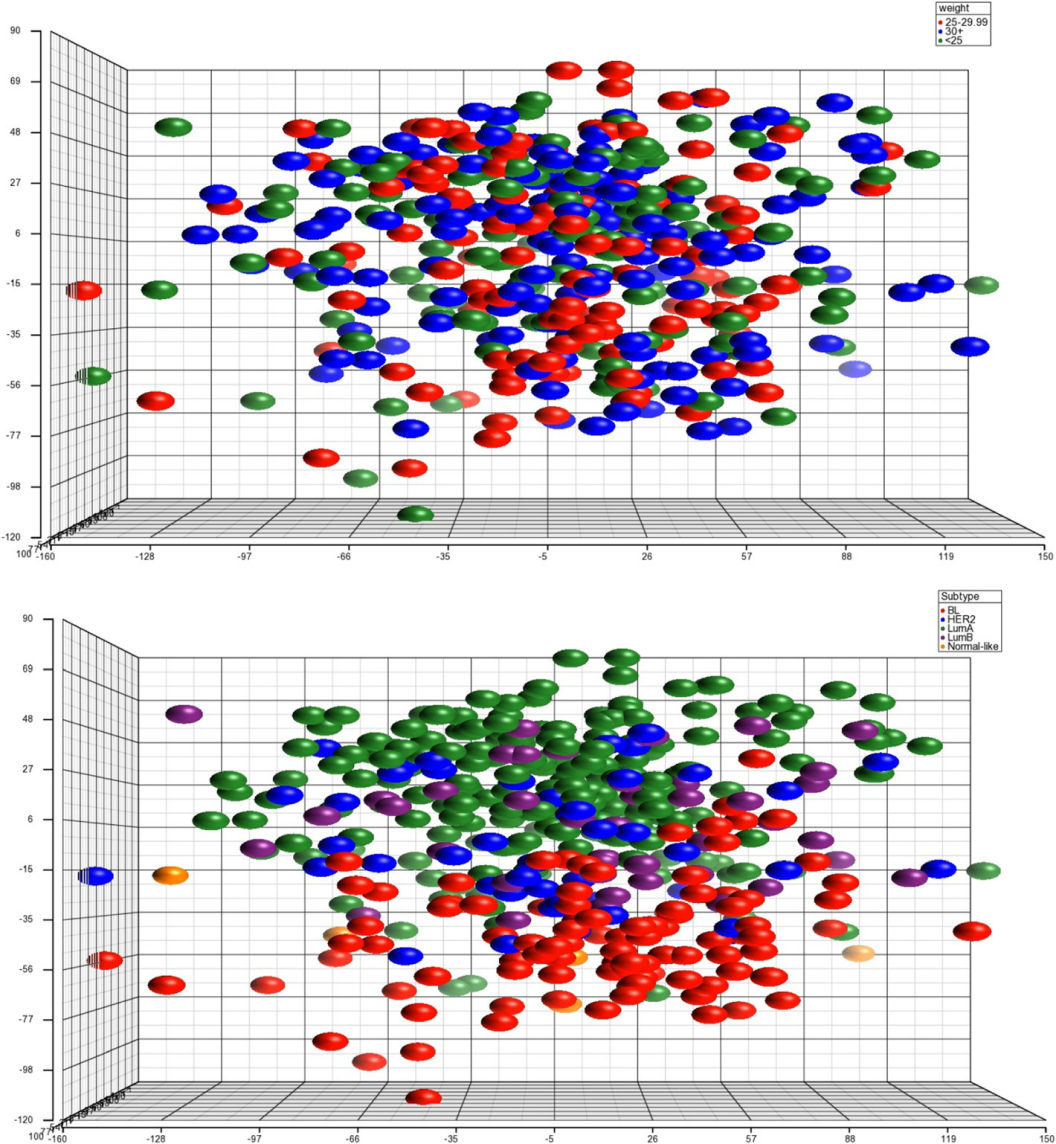
PCA of gene expression from 405 primary tumor samples. Plot on the top is colored by BMI groups with no obvious clusters detected. Plot on the left is colored by subtype and demonstrates grouping of the samples by subtype

**Fig. 2:**
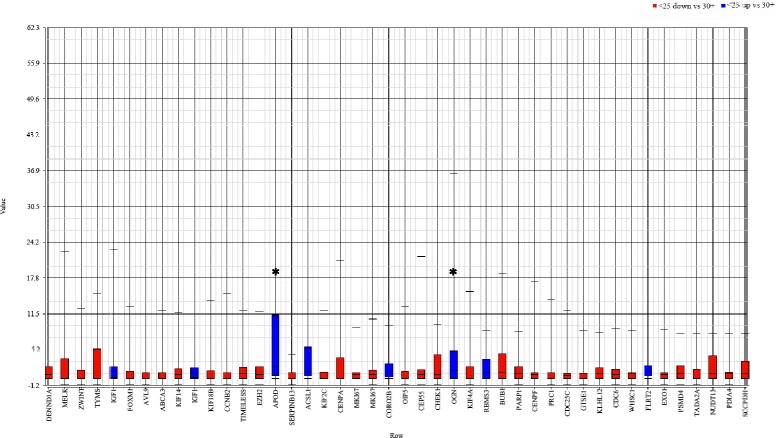
Box and whiskers plot of differential gene expression in tumors from normal weight and obese women. The highest fold-differences were detected for APOD and OGN with 2.36- and 2.05-fold higher expression in tumors from obese compared to normal weight women; MELK demonstrated the highest increase (1.71-fold) in expression in normal compared to obese women

PCA did not effectively cluster samples by BMI (Fig. 1). No differentially expressed genes were detected between BMI groups in the initial analysis which included all tumor subtypes or when comparing obese to non-obese patients. While PCA did not effectively discriminate tumors by BMI, tumors did cluster by intrinsic subtype. Thus, to determine whether the inclusion of a heterogeneous group of tumors was masking significant gene expression differences, analyses were performed within intrinsic subtypes. No differences were detected for luminal B (*n* = 43), HER2-enriched (*n* = 48) or basal-like (*n* = 99) tumors; however, 44 probes from 42 genes were differentially expressed by BMI category (Table 2; Fig. 2) within the luminal A subtype (*n* = 209). These differentially expressed genes are associated with a number of pathways involved in tumorigenesis, such as cell cycle control, mTOR and p53 signaling and DNA repair (Table 3).

**Table 2:** Genes differentially expressed in tumors from normal-weight compared to obese women. Genes in bold remained statistically significant when age at diagnosis and self-described ethnicity were included with BMI in ANOVA

		All BMI groups^a^	Normal-weight vs obese	
Gene Symbol	Probe	*P*-value	*P*-value	Fold-change (normal-weight/obese)
ABCA3	204343_at	4.5E-05	9.8E-06	0.66
ACSL1	201963_at	0.0001	3.0E-05	1.48
APOD	201525_at	0.0002	2.3E-05	2.36
AVL9	212471_at	4.9E-05	9.7E-06	0.82
BUB1	209642_at	1.6E-05	6.5E-05	0.64
CCNB2	202705_at	2.8E-05	1.4E-05	0.64
CDC25C	205167_s_at	0.0002	7.4E-05	0.67
CDC6	203968_s_at	0.0002	8.5E-05	0.72
CENPA	204962_s_at	8.9E-05	3.2E-05	0.59
CENPF	207828_s_at	2.3E-05	6.7E-05	0.65
CEP55	218542_at	7.6E-05	5.1E-05	0.60
CHEK1	205394_at	0.0002	5.1E-05	0.70
CORO2B	209789_at	0.0002	3.9E-05	1.27
DENND1A	219763_at	3.3E-07	5.0E-08	0.66
EXO1	204603_at	0.0003	8.8E-05	0.80
EZH2	203358_s_at	3.2E-05	1.5E-05	0.68
FLRT2	204359_at	0.0005	8.6E-05	1.60
FOXM1	202580_x_at	1.2E-05	8.9E-06	0.67
GTSE1	204317_at	0.0003	7.7E-05	0.83
IGF1	209542_x_at	2.4E-05	6.2E-06	1.75
	211577_s_at	3.5E-05	1.0E-06	1.72
KIF14	206364_at	1.5E-05	1.0E-05	0.70
KIF18B	222039_at	9.1E-06	1.1E-05	0.67
KIF2C	209408_at	0.0002	3.0E-05	0.66
KIF4A	218355_at	0.0001	5.5E-05	0.64
KLHL12	219931_s_at	0.0005	8.4E-05	0.79
MELK	204825_at	4.0E-06	2.2E-06	0.58
MKI67	212022_s_at	0.0007	3.5E-05	0.69
	212021_s_at	0.0002	3.4E-05	0.71
NUDT13	214136_at	0.0005	9.3E-05	0.74
OGN	218730_s_at	0.0003	5.4E-05	2.05
OIP5	213599_at	7.5E-05	4.5E-05	0.67
PARP1	208644_at	0.0004	6.7E-05	0.81
PDIA4	211048_s_at	0.0005	9.5E-05	0.80
PRC1	218009_s_at	0.0002	7.3E-05	0.66
PSMD4	200882_s_at	0.0005	9.1E-05	0.83
RBMS3	206767_at	0.0003	5.9E-05	1.28
SCCPDH	201826_s_at	0.0005	9.8E-05	0.73
SERPINB13	216258_s_at	3.9E-05	2.6E-05	0.93
TADA2A	209938_at	0.0005	9.1E-05	0.84
TIMELESS	203046_s_at	1.1E-05	1.5E-05	0.75
TYMS	202589_at	6.5E-06	3.9E-06	0.65
WHSC1	209054_s_at	0.0003	8.6E-05	0.81
ZWINT	204026_s_at	8.6E-06	3.1E-06	0.67

^a^Genes that differed significantly in expression levels when ANOVA was performed across normal weight, overweight and obese groups

**Table 3:** Differentially regulated pathways between tumors from normal-weight and obese women

KEGG pathway	Enrichment score	Enrichment P-value	% Genes present in pathway
Cell cycle	10.9	1.8E-005	4.3
p53 signaling pathway	10.4	3.1E-005	6.5
Progesterone-mediated oocyte maturation	9.6	7.0E-005	5.3
Oocyte meiosis	8.7	0.0002	4.2
One carbon pool by folate	3.1	0.0456	6.7
Mismatch repair	2.7	0.0692	4.3
Base excision repair	2.4	0.0951	3.1
Transcriptional misregulation in cancer	2.3	0.0962	1.2
ABC transporters	2.2	0.1064	2.8
Aldosterone-regulated sodium reabsorption	2.1	0.1175	2.5
Fatty acid metabolism	2.1	0.1231	2.4
Lysine degradation	2.1	0.1258	2.3
Proteasome	2.1	0.1285	2.4
mTOR signaling pathway	2.0	0.1394	2.1

## Discussion

Worldwide obesity rates are increasing at an alarming rate [29] and in the United States, >50 % of adults are expected to be obese by 2030 [4]. Given the poor prognosis of obese women with breast cancer, improved understanding of how obesity impacts survival is critical. Identification of molecular profiles in invasive breast carcinomas that correlate with obesity would allow for development of targeted therapeutics or risk reduction strategies that could improve outcomes in obese women. In this study, we detected 42 unique genes that were differentially expressed in luminal A breast tumors from normal-weight compared to obese women. Tumors from overweight patients did not differ significantly from those in normal-weight or obese women.

To our knowledge, this is the first study to identify transcriptomic changes associated with obesity in epithelial cells of luminal A tumors. Kwan et al. evaluated the effects of BMI in a set of 1,676 early-stage tumors where intrinsic subtype was assigned using the PAM50 qRT-PCR assay [11] and found that high obesity (BMI ≥ 35) was associated with decreased expression of ESR1 and increased expression of proliferation genes. Of the 10 proliferation genes assayed by Kwan et al., four (CENPF, CEP55, MK167 and KIF2C) were also expressed at significantly higher levels in tumors from obese compared to normal-weight patients in our study.

Fuentes-Mattei et al. performed microarray-based transcriptome analysis in ER+ tumors and identified 112 genes differentially expressed between non-obese and obese patients. Gene enrichment analysis detected significant alterations in the AKT-target and epithelial-mesenchymal transition pathways. Activation of the AKT/mTOR pathway was also detected in tumors from obese mice [30]. Although none of the differentially expressed genes from our study were also in the study from Fuentes-Mattei et al., pathway enrichment analysis of our differentially expressed genes also revealed alterations in the mTOR signaling pathway.

A transcriptomic signature of obesity encompassing 662 differentially expressed genes was previously reported based on tumor biopsy specimens from 103 female patients with locally advanced breast cancer enrolled in neoadjuvant studies, regardless of ER status [31]. Gene annotation enrichment analysis detected an expression signature overrepresented by genes involved in regulation of transcription and nucleus that was associated with shorter time to metastasis in two public data sets; however, no correlation was detected in four other databases. Of note, a significantly higher proportion of African Americans were obese compared to normal or overweight, and a number of differentially expressed probes in their dataset, including 205048_s_at (PSPHL), 206777_s_at (CRYBB2P1) and 212777_at (SOS1), are known to be differentially expressed in a variety of tissue types between African Americans and European Americans [32–39]. Inclusion of late-stage tumors not stratified by subtype or ER status, in combination with confounding gene expression results attributable to genetic ancestry, may have affected the ability to detect transcriptome changes associated with BMI.

A critical difference between our data and other reports is our use of laser microdissection to isolate tumor cells while samples from the other studies were comprised of 15–30 % stromal cells. Breast adipose tissue serves as fuel for tumor growth, recruits macrophages and stimulates an inflammatory response [40]. Data from our laboratory demonstrated that tumor-adjacent adipose has an altered inflammatory response and increased immunotolerance [41] and recent data demonstrates that co-culturing of ER+ breast cancer cells with adipose stromal/stem cells from obese women enhanced proliferation of the breast cancer cells, and these breast cancer cells demonstrated increased epithelial-mesenchymal transition and expression of metastasis genes [42]. Thus, while laser microdissection of tumor cells may have provided a molecular portrait of gene expression in the tumor epithelia, studies that allowed for a significant proportion of stromal cells, including adipose, may have detected alterations associated with excess adiposity that are present in the tumor microenvironment.

Limitations of this study include lack of long-term follow-up and treatment information as well as limited sample sizes for the non-luminal A subtypes. Samples were collected 2001–2011, thus long-term outcome information was not available for all patients. Given that luminal A tumors have a longer time to relapse (5–15 years) than other subtypes [43], differences in long-term mortality by BMI may not be detected. In conjunction, these patients were treated at WRNMMC, a Department of Defense military hospital. Although all patients within this equal-access health care system are provided standard health care, it was not possible to determine whether treatment regimens were equivalent for obese women, or if any women received agents such as mTOR inhibitors that may be more effective in treating obese women with luminal A breast tumors. Finally, lack of differentially expressed genes in the non-luminal A subtypes, especially luminal B tumors, may reflect small sample sizes. Power analysis demonstrated that to detect ≥1.5 fold expression level differences with 80 % power, a minimum of 43 patients in each BMI group would be needed. Within the luminal A subtype, 68, 64 and 77 tumors were from normal weight, overweight and obese women, respectively; across the other subtypes there were a total of 43, 48 and 99 luminal B, HER2-enriched and basal-like tumors total.

## Conclusion

Excess adiposity does not affect all breast tumors equally; rather, differential gene expression by BMI was restricted to luminal A tumors. Alterations in pathways associated with cell cycle control, mTOR and p53 signaling, and fatty acid metabolism may explain the less favorable outcomes associated with obesity. In addition, detection of alterations in these pathways allows for the use of agents such as mTOR inhibitors to more effectively treat obese women with luminal A tumors and decrease outcome disparities.

## Availability of data and materials

Microarray data has been deposited in GEO (http://www.ncbi.nlm.nih.gov/geo/; Accession number: GSE78958).

## Abbreviations

BMI: body mass index
ER: estrogen receptor
HRT: hormone replacement therapy
